# Comparison of the effect of licorice and chlorhexidine mouthwash on the oral health of intubated patients in the intensive care unit

**DOI:** 10.1186/s12903-024-04456-7

**Published:** 2024-06-12

**Authors:** Khodayar Oshvandi, Reza Faghih Lotfi, Azim Azizi, Leili Tapak, Amir Larki-Harchegani

**Affiliations:** 1 Department of Medical Surgical Nursing, School of Nursing and Midwifery, Hamadan University of Medical Sciences, Hamadan, Iran; 2 Student M.Sc. in Critical Care Nursing, School of Nursing and Midwifery, Hamadan University of Medical Sciences, Hamadan, Iran; 3 Chronic Diseases (Home Care) Research Center, Department of Medical Surgical Nursing, School of Nursing and Midwifery, Hamadan University of Medical Sciences, Hamadan, Iran; 4 Department of Biostatistics, School of Public Health and Modeling of Noncommunicable Diseases Research Center, Hamadan University of Medical Sciences, Hamadan, Iran; 5 Department of Pharmacology and Toxicology, Medicinal Plants and Natural Products Research Center, School of Pharmacy, Hamadan University of Medical Sciences, Hamadan, Iran

**Keywords:** Mouthwashes, Glycyrrhiza, Chlorhexidine, Oral health, Intensive care units, Nurses

## Abstract

**Background:**

Chlorhexidine mouthwash is a common oral hygiene product used in intensive care units, but it may have some adverse effects. Licorice, a natural herb, could be a potential alternative to chlorhexidine. However, the effect of licorice mouthwash on the oral health of intubated patients has not been studied yet. Therefore, this study aimed to compare the effects of licorice and chlorhexidine mouthwash on the oral health of intubated patients.

**Methods:**

This was a triple-blind clinical trial. The sample included 130 intubated patients admitted to an intensive care unit in Iran. The samples were selected by convenience sampling and randomly assigned to two groups: A and B. In group A, the main researcher applied 15 ml of 0.2% chlorhexidine mouthwash after each brushing (twice a day for 5.5 days) and suctioned it after 30 s. In group B, 20% licorice mouthwash was used instead of chlorhexidine. The demographic information questionnaire and the Beck Oral Assessment Scale (BOAS) were completed by one of the nurses before and on the sixth day of the study.

**Results:**

Finally, 60 patients in each group completed the study. There was no significant difference between the groups in terms of demographic variables or oral health before the intervention (*P* > 0.05). The oral health of patients in both the chlorhexidine and liquorice mouthwash groups improved significantly after the intervention (*P* < 0.05). However, there was no significant difference in oral health between the two groups at postintervention (*P* = 0.06).

**Conclusion:**

The results demonstrated that both mouthwashes exerted a comparable effect on dental and oral health. However, the chlorhexidine mouthwash showed a greater impact on the reduction of dental plaque and the thinning of saliva compared to licorice mouthwash. In essential cases, licorice mouthwash can be employed as an alternative to chlorhexidine.

## Background

Oral and dental health are among the most important factors affecting the general health of patients admitted to intensive care units (ICUs) [1]. Maintaining oral and dental hygiene in ICUs can help prevent oral and dental infections, ventilator-associated pneumonia, gingivitis, oral bleeding, bad breath and pain [2–4]. One of the main responsibilities of nurses is to provide and improve oral hygiene in these units [5]. However, patients with endotracheal tubes admitted to ICUs are not able to take care of their own mouths, and nurses also face challenges in providing effective oral care to these patients due to fear of displacing or removing endotracheal tubes and gastric tube health [6, 7].

The best method of maintaining oral and dental hygiene in ICUs is still a matter of debate. Many previous studies have shown that using antibacterial mouthwashes such as chlorhexidine can help reduce dental plaque, gingivitis and ventilator-associated pneumonia [8–10], but these mouthwashes may also have side effects such as tooth discoloration, unpleasant taste, dry mouth and negative systemic effects [11, 12].

On the other hand, some medicinal plants, such as liquorice, have antibacterial, anti-inflammatory, antiulcer and immunostimulatory properties that can be beneficial for the oral health of patients [13–15]. Although licorice is a natural substance, it can cause adverse effects, especially when consumed in large quantities or over a prolonged period. This issue is of particular concern for ICU patients, who are more prone to negative side effects. Glycyrrhizin, a compound in licorice root, can lead to complications such as increased blood pressure, hypokalemia, edema, fatigue, and dysrhythmia. In severe cases, especially among individuals with pre-existing cardiac, renal, or hypertensive conditions, the consumption of significant amounts of licorice (in excess of 5 g daily) for several weeks may result in serious side effects, including myocardial infarction [16, 17]. Therefore, it is imperative for healthcare professionals to be cognizant of these potential risks when considering the administration of licorice to patients, particularly in the sensitive environment of an ICU.

The results of the study by Melania et al. showed that licorice mouthwash had a significant effect on reducing gingival inflammation in patients with gingivitis, suggesting that this product could serve as an alternative treatment for gum infections [14]. Furthermore, the study conducted by Firouzian at al. demonstrated that the use of licorice extract could prevent the occurrence of gastrointestinal bleeding and ventilator-associated pneumonia. These effects are attributed to the antibacterial, anti-reflux, and anti-inflammatory properties of the extract in patients who have endotracheal tubes and are hospitalized in the intensive care unit [18].

Although licorice has been proposed as an alternative option for chlorhexidine in the treatment of gum infections, its effect on the oral health of patients with endotracheal tubes and a reduced level of consciousness in ICUs has not been investigated [15, 19]. On the other hand, replacing chlorhexidine with an herbal product with low side effects and low cost can lead to reduced complications and treatment costs and increased patient satisfaction [18]. Therefore, the research question is whether licorice mouthwash, which is an herbal product, can be used as a suitable alternative to chlorhexidine mouthwash for improving the oral health of patients with endotracheal tubes in ICUs.

## Methods

### Design

This study was a clinical trial with a two-group pretest-posttest design and three blinders, in which the main researcher, patients (due to reduced level of consciousness), evaluator and statistical analyzer were unaware of the study groups.

This study was designed as an equivalence trial to compare the effects of licorice and chlorhexidine mouthwash on the oral health of mechanically ventilated patients in the intensive care unit. The aim was to demonstrate that licorice mouthwash is not inferior to chlorhexidine mouthwash and could be a suitable alternative. The equivalence margin was determined based on clinical judgment and statistical considerations, and the sample size was calculated to ensure sufficient power to confirm the hypothesis of equivalence.

### Sampling and setting

This study involved the participation of intubated patients admitted to the Qaem ICU of Shahid Beheshti Hospital in Hamadan, located in northwest Iran, from February 2022 to September 2023. Study inclusion required individuals aged 18 to 65 with an oral endotracheal tube, airway, or nasogastric tube, without coagulation and immune disorders, pulmonary infections, sepsis, allergies to study substances, pregnancy, or prior antibiotic use. Exclusion applied to those with hypokalemia, smoking history, coronary artery disease, heart failure, renal failure, or hypertension. Exclusion criteria included the death or transfer of patients, premature removal of the endotracheal tube, withdrawal from the study by the individual or legal guardian, or the occurrence of complications from licorice consumption such as renal failure, hypertension, hypokalemia, and cardiac dysrhythmia [20]. Considering a confidence level of 95% and a power of 80%, a mean difference of 0.5, a variance of 0.93 and a variance of 0.92 [19], and a 20% probability of sample dropout, the sample size in each group was estimated to be at least 65 persons.

### Recruitment and allocation

**Fig. 1:**
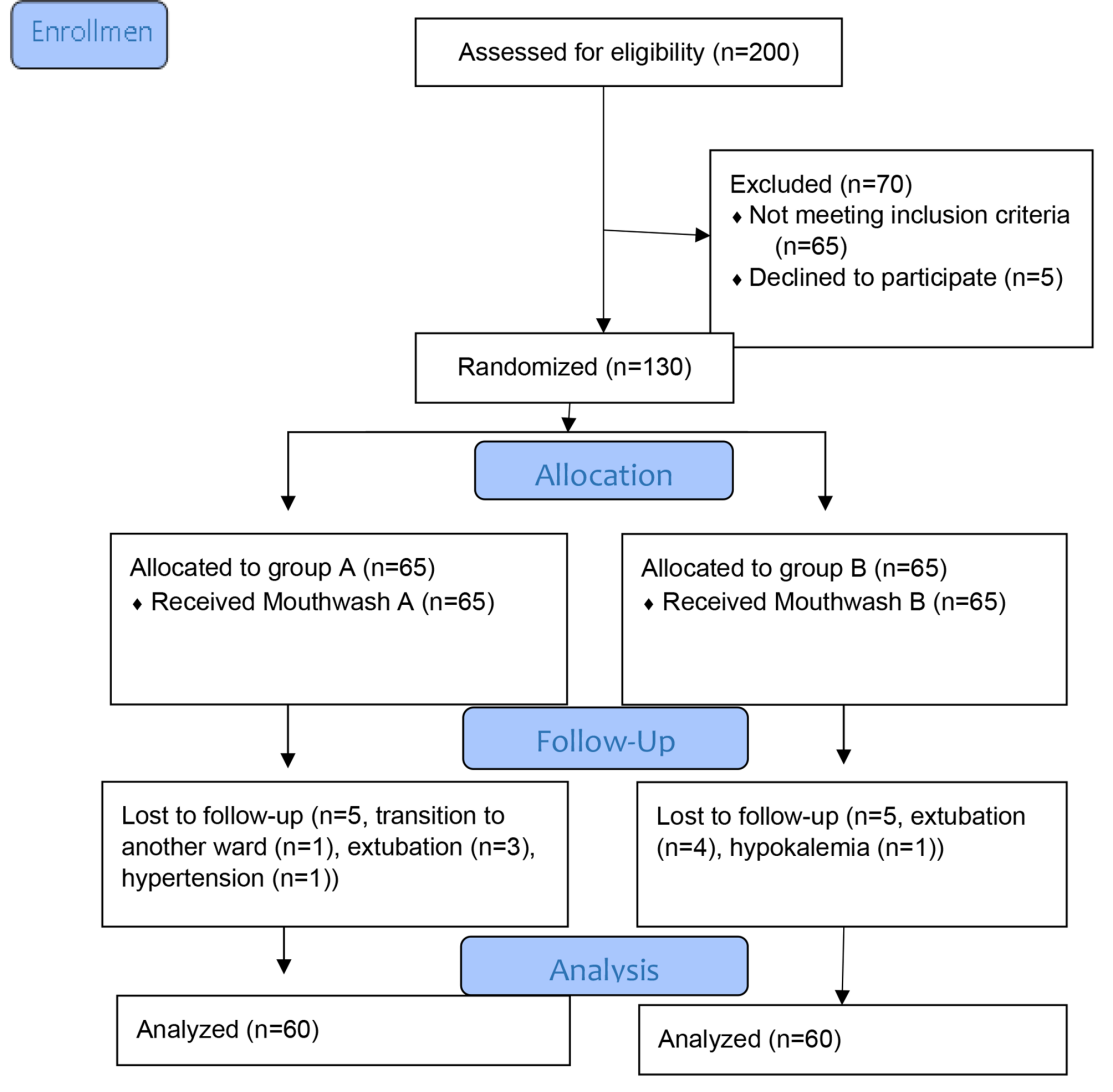
Study flow diagram based on the Consort statement of the year 2010

First, patients who met the inclusion criteria were selected by convenience sampling, and then, sequentially numbered, sealed, opaque envelopes were used. Patients were allocated to two groups: group A (0.2% chlorhexidine digluconate group) and group B (20% liquorice group). In each of the chlorhexidine and liquorice mouthwash groups, 65 patients entered the study, but 5 patients from each group dropped out; thus, 60 patients completed the study (Fig. 1).

### Outcome measures

The data collection tools included a demographic and clinical characteristics questionnaire of patients and the Beck Oral Assessment Scale (BOAS).

### Demographic and clinical characteristics

The demographic and clinical characteristics of the patients included age, sex, body mass index, method of nutrition, GCS score, FOUR score, cause of hospitalization, underlying disease, number of teeth, blood pressure and heart rate.

### Beck oral assessment scale (BOAS)

We used the Beck Oral Assessment Scale (BOAS) to assess oral health. This scale has five subscales: lips, mucosa and gums, teeth, tongue and saliva. Each subscale is graded from one to four based on health status. The total score ranges from five to 20, where five indicates no disorder, six to 10 indicates mild disorder, 11 to 15 indicates moderate disorder and 16 to 20 indicates severe disorder [21, 22]. This scale was translated and validated by Safarabadi et al. (2012) in Iran [21]. They assessed and confirmed its validity using face validity, content validity and construct validity. They also confirmed its reliability using the interrater reliability method with a kappa coefficient of 0.92 [22]. In this study, the kappa coefficient for each dimension was estimated to range from 0.88 to 0.94, and the kappa coefficient of the total questionnaire was 0.91.

### Procedures

In Group A, a 0.2% chlorhexidine digluconate mouthwash produced by Shahre Daru Company (http://www.shahredaru.com/en/contact) in Tehran, Iran, was employed. In contrast, Group B used a 20% liquorice mouthwash solution. This solution was formulated to achieve the minimum inhibitory and bactericidal concentrations required to target periodontal pathogens, resulting in a 20% (w/v) aqueous extract of licorice root. Mouthwash chlorhexidine digluconate was added by a pharmacist to a container labeled A, and the mouthwash liquorice was added to another container labeled B and was given to the main researcher; until the end of the study, only the pharmacist knew about their contents.

Before the intervention, one of the morning shift nurses (nursing expert) who was unaware of the study groups completed the demographic and clinical characteristics questionnaire and the Beck Oral Assessment Scale for both groups. The nurse used information from the file and assessed the oral health and teeth of the participants using a flashlight and a special mirror for dentistry. The nurse received the necessary training under the supervision of an oral health specialist. In both groups, before performing oral care, the cuff pressure was adjusted to between 25 and 20 mmHg using a special manometer to ensure its appropriateness. The headboard was raised to 30 degrees, and the patient’s head was turned to one side. A towel was spread around the chin and head of the patient. A receiver was placed next to the patient’s mouth. Twice a day (morning and evening), a special soft toothbrush for children was used to brush all areas of the mouth, including the internal and external surfaces of the teeth (with circular movements), the gums and the tongue (with movements from back to front), without using toothpaste. After brushing, the oral cavity was rinsed with normal saline and suctioned. During each brushing, the airway of the samples under study was removed, cleaned and placed back in the patient’s mouth after using mouthwash. All these procedures in both groups were the same and were performed by the main researcher.

Intervention group A: In group A, in addition to the intervention described above, all parts of the mouth were rinsed with a syringe containing 15 ml of mouthwash solution with label A (chlorhexidine digluconate 0.2%) immediately after each brushing (twice a day at 12-hour intervals) and suctioned after 30 s. The intervention lasted for 5.5 days. Intervention group B: In group B, the interventions were the same as those in group A, except that mouthwash with label B (20% licorice mouthwash) was used instead of mouthwash A. Finally, on the morning of the sixth day, after the oral care of the patients by the main researcher following the research protocol, oral health was assessed and recorded by the same nurse who used the Beck scale before the intervention.

### Data analysis

The data were analyzed using SPSS software version 16, and descriptive and inferential statistics, including the Kolmogorov‒Smirnov test (to check the normality of the data distribution), chi‒square tests and Fisher’s exact tests (to compare categorical variables), independent t tests (to compare continuous variables between two groups) and paired t tests (to compare continuous variables within each group), were used. The significance level was set at less than 0.05.

## Results

The results of the study showed that there was no significant difference between the chlorhexidine and licorice mouthwash groups in terms of demographic and clinical variables before the intervention. These variables included age, number of teeth, frequency of brushing before admission, Glasgow Coma Scale score, systolic and diastolic blood pressure, heart rate, respiratory rate, SPO2, temperature, sex, cause of hospitalization, underlying disease, brushing before admission, use of antacid, and method of nutrition (*p* > 0.05). Therefore, the two groups were similar in terms of these variables (Table 1).

**Table 1:** Comparison of the demographic characteristics of the ventilated patients in the licorice and chlorhexidine, mouthwash groups

Variables		Licorice mouthwash group(*n* = 60)	Chlorhexidine mouthwash group (*n* = 60)	*P*-value*
		M ± sd	M ± sd	
Age (year)		53.28 ± 11.49	51.30 ± 11.59	0.349
Number of teeth		24.86 ± 2.87	25.70 ± 3.11	0.130
Daily brushing frequency		1.18 ± 0.792	1.05 ± 0.910	0.394
Four scale		12.72 ± 2.38	12.28 ± 1.20	0.212
Glasgow coma scale		10.35 ± 2.83	9.88 ± 1.71	0.132
Systolic blood pressure (mmHg)		119.15 ± 12.01	116.70 ± 15.52	0.335
Diastolic blood pressure (mmHg)		68.12 ± 9.88	70.23 ± 13.29	0.147
Heart rate		82.45 ± 7.19	85.12 ± 10.01	0.09
Respiration rate		20.93 ± 1.20	20.47 ± 2.66	0.211
Spo2		94.27 ± 2.122	93.73 ± 2.84	0.246
Temperature		37.11 ± 0.276	37.05 ± 0.14	0.149
Variables		Frequency (%)	Frequency (%)	*P*-value
Gender	Male	28 (46.7)	25 (41.7)	0.581**
	Female	32 (53.3)	35 (58.3)	
Brushing teeth before admission	Yes	47 (78.3)	42 (70.0)	0.297**
	No	13 (21.7)	18 (30.0)	
Use of antacid drug	Yes	53 (88.3)	57 (95.0)	0.322***
	No	7 (11.7)	3 (5.0)	
Feeding method	NPO	3 (5.0)	5 (8.3)	0.717***
	Gavage	57 (95.0)	55 (91.7)	
Cause of hospitalization	Surgery	14(23.3)	11 (18.3)	0.960***
	Trauma	13 (21.7)	15 (25.0)	
	Decreased level of consciousness	9(15.0)	8 (13.3)	
	Breathing problems	13 (21.7)	10 (16.7)	
	Deep Venus thrombosis	3 (5.0)	3 (5.0)	
	Liver failure	1 (1.7)	1 (1.7)	
	Digestive bleeding	3 (5.0)	5 (8.3)	
	Weakness	2 (3.3)	2 (3.3)	
	Anemia	0 (0.0)	1 (1.7)	
	Other	3 (5.0)	7 (11.7)	
Underlying disease	Diabetes	5 (8.3)	6 (10.0)	0.977***
	Liver diseases	2 (3.3)	1 (1.7)	
	Respiratory diseases	8 (13.3)	6 (10.0)	
	Hyperlipidemia	4 (6.7)	4 (6.7)	
	Stomach reflux	7 (11.7)	8 (13.3)	
	Stroke	4 (6.7)	2 (3.3)	
	Kidney diseases	2 (3.3)	4 (6.7)	
	More than one case	12 (20.0)	14(23.3)	
	No previous illness	16 (26.7)	15 (25.0)	

*Independent *t* test, ** Chi square, *** Fisher Exact Test

The results of paired t tests showed that the mean total oral health score and its dimensions (lips, gums and oral mucosa, tongue, teeth, and saliva) in both groups (licorice and chlorhexidine mouthwash) decreased after the intervention compared to before the intervention, and this decrease was statistically significant (*p* < 0.001). This means that oral health and its dimensions improved after the intervention in both groups. Additionally, the results of the independent t test showed that there was no significant difference between the two groups receiving licorice or chlorhexidine mouthwash in terms of mean oral health and its dimensions before the intervention (*p* > 0.05). However, after the intervention, there was a significant difference between the two groups in terms of the dental health and saliva dimensions (*p* < 0.05), so oral health in these dimensions was greater in the chlorhexidine mouthwash group than in the liquorice mouthwash group. However, for the other dimensions (lips, gums and oral mucosa, tongue) and total oral health score, there was no significant difference between the two groups (*p* > 0.05) (Table 2).

**Table 2:** Comparison of the average oral health and its subscales between-within the two groups before and after the intervention

Variables	Time of evaluation	licorice mouthwash group (*n* = 60)	Chlorhexidine mouthwash group (*n* = 60)	statistic	*p*-value*
		M ± SD	M ± SD		
Lips	Before	2.53 ± 0.676	2.58 ± 0.619	0.423	0.673
	After	1.90 ± 0.399	1.93 ± 0.548	0.381	0.704
	statistic	8.898	9.205		
	*p*-value**	˂0.001	˂0.001		
Gums and oral mucosa	Before	2.53 ± 0.812	2.73 ± 0.821	1.342	0.182
	After	1.83 ± 0.642	1.93 ± 0.362	1.051	0.296
	statistic	7.294	8.211		
	*p*-value**	˂0.001	˂0.001		
Tung	Before	2.57 ± 0.851	2.58 ± 0.671	0.119	0.905
	After	2.07 ± 0.634	1.98 ± 0.748	0.658	0.512
	statistic	5.028	8.322		
	*p*-value**	˂0.001	˂0.001		
Teeth	Before	2.90 ± 0.706	2.93 ± 0.634	0.272	0.786
	After	2.27 ± 0.516	2.00 ± 0.521	-2.817	0.006
	statistic	8.898	11.399		
	*p*-value**	˂0.001	˂0.001		
Saliva	Before	2.83 ± 0.740	2.95 ± 0.723	0.873	0.384
	After	2.23 ± 0.563	2.00 ± 0.451	-2.504	0.014
	statistic	7.543	11.824		
	*p*-value**	˂0.001	˂0.001		
Total score of oral health	Before	13.37 ± 2.176	13.82 ± 1.987	1.180	0.240
	After	10.30 ± 1.857	9.79 ± 1.403	-1.889	0.061
	statistic	17.915	24.760		
	*p*-value**	˂0.001	˂0.001		

* Independent *t* test, **paired *t* test

## Discussion

This study aimed to compare the effects of licorice and chlorhexidine mouthwash on the oral health of intubated patients in the intensive care unit. The results, obtained using paired t tests, showed that in both the chlorhexidine and liquorice mouthwash groups, the oral health of intubated patients in the intensive care unit and its dimensions improved compared to before the study. These findings are consistent with those of Melaniya et al. (2019), who demonstrated that in intragroup comparisons, both the plaque index and gingival index in the chlorhexidine and liquorice mouthwash groups decreased significantly after the intervention compared to before the intervention [23]. In addition, the results of Jabbari Ghanati et al. (2018) showed that after four days of intervention in the chlorhexidine group, oral health improved significantly compared to that before the intervention [8]. The results of the present study are also consistent with the results of Safarabadi et al. (2012) [22], Desai et al. (2023) [24], and Jain et al. (2017) [19]. In contrast to the present study, Goltashin et al. showed that glycyrrhizin added to toothpaste had no effect on the plaque index or gingival index [25]. This difference may be due to the inappropriate concentration of glycyrrhizin, the reduction in its effect in combination with chemical substances present in toothpaste or the increase in its antimicrobial effect due to other compounds present in liquorice.

Before the intervention, the mean health score of both groups was between 11 and 15, so it can be said that, on average, the participants had moderate disorders in oral and dental health [22]. Although nonsmoking and nonaddiction to drugs were among the criteria for inclusion in the study, the results of this study showed that oral and dental health of hospitalized patients in intensive care units was not desirable at admission. This finding is consistent with the findings of Munro and Grap, who stated that the oral and dental health of patients who are hospitalized in intensive care units may be weak before admission [26]. However, after the intervention, the mean Beck scale score in both groups was lower than 11. Therefore, regardless of the type of intervention, their mean oral health status score was in the range of mild disorders [22]. Therefore, performing a precise program of oral care by toothbrushing and mouthwashing improved the oral health status of both groups. In addition, the results of this study show the need for more attention to oral and dental health at the community level.

After the intervention, compared with those in the liquorice mouthwash group, oral health in terms of dental health and saliva in the chlorhexidine mouthwash group improved. However, for the other dimensions and the total score of oral health, no significant difference was observed between the two groups. Therefore, it can be concluded that the consumption of chlorhexidine mouthwash was more effective than the consumption of licorice mouthwash in reducing dental plaque and reducing saliva concentration and viscosity. The change in saliva quality to watery and very watery states in the chlorhexidine mouthwash group may be due to a reduction in the oral microbial load and stimulation of more saliva secretion by chlorhexidine than by licorice mouthwash. However, generally, using both mouthwashes had a similar effect on improving the overall oral health of patients with endotracheal tubes hospitalized in the intensive care unit.

The possible causes of increased oral health in the liquorice group can be attributed to the anti-inflammatory, antiviral, antiallergic, antimicrobial, antitussive, mucolytic and immunostimulatory properties of this substance [13–15, 27]. Microbial activity is inhibited by its presence. These compounds prevent the growth of microorganisms. The presence of secondary metabolites such as alkaloids, saponins, flavonoids and terpenoids also contributes to antibacterial activity [28]. A reduction in bacterial gene expression, bacterial growth and bacterial toxin production are some of the mechanisms by which this substance functions [28, 29].

This finding is consistent with the study of Desai et al. (2023), who concluded that liquorice mouthwash can be substituted for chlorhexidine mouthwash [24]. Although the variables measured and the tools used in the studies differed, the results were similar to those of the present study. However, this was a triple-blind study, which minimized the influence of external factors such as the evaluator’s effect or the presence of the researcher and increased the accuracy of the results. However, the results of this study are inconsistent with those of Jin et al., who reported that the gingival inflammation index was greater in the chlorhexidine mouthwash group than in the liquorice group (*p* < 0.05). In Jin’s study, the concentration of liquorice mouthwash was not mentioned [19]. The difference in results may be due to the longer duration of intervention in Jin et al.‘s study, the difference in the variable evaluated, and the difference in the concentration of mouthwashes used.

The Food and Drug Administration has labeled licorice as a safe compound. Licorice is “generally recognized as safe”, and it is suggested that it can be used if there is no sensitivity [30, 31]. According to a World Health Organization report, 100 mg per day licorice can be consumed safely without side effects [32].

The results of this equivalence trial indicated that licorice mouthwash could be considered as an alternative to chlorhexidine mouthwash. Both mouthwashes significantly improved the oral health of the patients, and no significant difference was observed in the outcomes between the two groups, confirming their equivalent efficacy. However, it should be noted that chlorhexidine mouthwash was more effective in reducing dental plaque and saliva concentration and viscosity. This factor may be important in selecting the appropriate mouthwash for different patients.

### Side effects of mouthwashes

To identify and mitigate the significant side effects associated with chlorhexidine and licorice mouthwashes, parameters such as blood pressure, heart rhythm, respiration, temperature, level of consciousness, potassium, sodium, and renal function were meticulously monitored and measured. In the chlorhexidine mouthwash group, an individual was diagnosed with elevated blood pressure, while in the licorice mouthwash group; a case of hypokalemia was identified, leading to the exclusion of the affected participant from the study. Considering that these solutions were suctioned away following oral rinsing, resulting in minimal systemic absorption, no other significant side effects were observed. Given the complex condition of the patients, these incidents cannot be directly and confidently attributed to the mouthwashes used.

### Strengths and limitations of the study

This study possesses several notable strengths, including its triple-blind design, the random allocation of participants into two distinct groups, the meticulous laboratory preparation of licorice mouthwash with antibacterial properties, the consistent and precise execution of interventions by the researcher, and the distinction of being the first study to compare the effects of chlorhexidine and licorice mouthwashes in hospitalized ICU patients. This was further enhanced by the engagement of external evaluators who were independent of the research team. However, the study also presents several limitations. These include the shortened duration of the intervention, which was necessitated by concerns regarding patient discharge and the potential loss of research samples; the oral health checklist was completed by an individual who was not a specialist in the field; and the lack of a proportional comparison between the concentrations of chlorhexidine and licorice mouthwashes, due to the differences in their antimicrobial killing concentrations.

## Conclusion

This clinical trial has established that licorice mouthwash is a feasible alternative to chlorhexidine for preserving oral hygiene in intubated patients within ICU environments. The results of the study indicate that both mouthwashes significantly enhanced oral health, with no substantial difference in their effectiveness. This suggests that licorice, owing to its herbal composition, might serve as an efficacious replacement for chlorhexidine. Licorice particularly highlights its potential as a safer choice for patients susceptible to adverse effects from chlorhexidine. Nonetheless, it is crucial to acknowledge that chlorhexidine exhibited a marginally superior performance in diminishing dental plaque and modifying salivary characteristics. Such a differentiation could assist healthcare practitioners in selecting the most suitable oral rinse, taking into account the specific requirements and circumstances of each patient. In summary, licorice mouthwash stands out as a promising contender to chlorhexidine, providing a harmonious blend of efficacy and safety for oral maintenance in ICU settings. It is recommended that the effect of these mouthwashes on oral microbial strains be examined using microbial culture in future studies.

## Data Availability

The data supporting the findings of this study will be available at https://isid.research.ac.ir/Khodayar_Oshvandi if needed.
